# Direct Oral Anticoagulants Use in Antiphospholipid Syndrome: Are These Drugs an Effective and Safe Alternative to Warfarin? A Systematic Review of the Literature: Comment

**DOI:** 10.1007/s11926-017-0675-3

**Published:** 2017-07-20

**Authors:** Hannah Cohen, Beverley J. Hunt, Maria Efthymiou, Ian J. Mackie, Munther Khamashta, David A. Isenberg, On behalf of the RAPS Trial Investigators

**Affiliations:** 1 Department of Haematology, University College London Hospitals NHS Foundation Trust, London, UK; 2 Haemostasis Research Unit, Department of Haematology, University College London, 51 Chenies Mews, London, WC1E 6HX UK; 3 Department of Thrombosis and Haemophilia, Guy’s and St Thomas’ Hospitals NHS Foundation Trust, London, UK; 4 Department of Haematology, King’s College London, London, UK; 5 Lupus Research Unit, Division of Women’s Health, King’s College London, London, UK; 6 Centre for Rheumatology, Division of Medicine, University College London, London, UK; 7 Department of Rheumatology, University College London Hospitals NHS Foundation Trust, London, UK

**Keywords:** Antiphospholipid syndrome, Venous thromboembolism, Rivaroxaban, Warfarin, Thrombin generation

## Abstract

We respond to comments by Dufrost et al. about the RAPS trial, in particular, showing that the trial did achieve its target sample size; pointing out that thrombin potential is not synonymous with overall thrombin generation; confirming that overall, no increased thrombotic risk was evident comparing rivaroxaban with warfarin; and that high-risk patients (28% were triple positive, representative of patients with venous thromboembolism requiring standard-intensity anticoagulation) were included; and clarifying our rationale for using a laboratory surrogate primary outcome measure instead of a clinical one.

## Commentary

We write to correct some inaccurate statements about the rivaroxaban in antiphospholipid syndrome (RAPS) trial in Dufrost et al.’s review [1, 2].

They indicate that the RAPS trial did not achieve the target sample size. The RAPS trial paper stated that 58 patients per group would need to be enrolled to ensure with 80% power that a two-sided 95% CI would exclude the non-inferiority threshold, assuming a common SD of 36%, one-sided significance level of 2.5% and 12% of patients who were not assessable for the primary outcome. It follows that the 54 and 56 patients analysed (i.e. which excluded 6 patients [5.2% of the total 116 recruited] who were not assessable), for the primary outcome measure in the rivaroxaban and warfarin groups, respectively, were sufficient to achieve the planned statistical power.

Second, Dufrost et al. stated “Overall, patients treated with rivaroxaban had a significant twofold-increased thrombin potential, suggesting a higher thrombotic risk. However, authors stated that no increased thrombotic risk was noticed in the rivaroxaban arm compared to standard-intensity warfarin because no clinical event occurred during the short follow-up (210 days).” The RAPS paper stated that when anticoagulation intensity was assessed by percentage change in ETP alone, rivaroxaban was inferior to warfarin in patients with antiphospholipid syndrome (APS) and previous venous thromboembolism. However, peak thrombin generation was lower with rivaroxaban and, therefore, the overall thrombogram indicated no difference in thrombotic risk. This conclusion is supported by in vivo coagulation activation marker concentrations being raised in only a few patients in both treatment groups. Additionally, no new thrombotic events were seen during 6 months of treatment.

The RAPS trial paper explained with regard to interpretation of the overall thrombogram: “Warfarin, therefore, affects all phases of thrombin generation equally, whereas rivaroxaban mainly affects the initiation and propagation of thrombin generation, leading to a delay in formation of the prothrombinase complex. As a result, the thrombin generation curve becomes protracted, which in turn lengthens the lag time and time to peak thrombin generation, and leads to greater ETP than would be expected for the degree of anticoagulation.” Our conclusions from the RAPS trial are supported by the independent expert comment [3], which stated “The endogenous thrombin potential (the parameter most frequently reported in the literature for calibrated automated thrombography), indicated inferiority of rivaroxaban. By contrast, peak thrombin concentrations, which Cohen and colleagues argue more accurately reflects thrombotic risk than endogenous thrombin potential, suggested non-inferiority, supporting their conclusion of non-inferiority of rivaroxaban.”

Further, Dufrost et al. imply that thrombin potential (correctly termed endogenous thrombin potential) is synonymous with overall thrombin generation. It is not. The thrombin generation curve (or thrombogram) is quantified in terms of the lag time, time to peak thrombin generation, peak thrombin generation, and endogenous thrombin potential (ETP), which is the area under the curve.

We explained our rationale for using a laboratory surrogate outcome measure as follows: “Rather, the trial was designed with a laboratory surrogate outcome measure to assess the mechanism of action of the interventions in these patients. A trial with a primary endpoint of recurrent thrombosis would require a sample of several thousand patients, which is unfeasible for patients with thrombotic APS, and a much longer follow-up period.”

Third, Dufrost et al. stated “Indeed, high-risk APS patients were not included” and “a low percentage of patients with triple positivity were included (25%).” The RAPS paper stated “28% of patients in RAPS had triple positivity.” We also stated “The RAPS trial had an intended selection bias because we excluded patients who had had venous thromboembolism (VTE) and developed recurrent events while taking standard-intensity anticoagulation (i.e. needing higher-intensity anticoagulation) and those with arterial events.” The proportion of triple positive patients included in RAPS is representative of VTE patients requiring standard-intensity anticoagulation in our APS population and consistent with the proportion in APS patients suggested in a large multicentre study [4].

In conclusion, we believe that the concerns raised by Dufrost et al. [2] are unfounded.
